# The Effect of Testosterone Replacement on Intramedullary, Inguinal and Visceral Fat in Ovariectomized Rats

**DOI:** 10.1055/s-0040-1701460

**Published:** 2020-01

**Authors:** Lorena Doretto da Silva, Juliana Mora Veridiano, Jussara Celi Conceição Oliveira, Anna Carolina Haddad Sayeg, Ana Maria Amaral Antonio Mader, Giuliana Petri, Bianca Bianco, César Eduardo Fernandes, Olga Maria Szymanski de Toledo, Luciano de Melo Pompei, Marcelo Luis Steiner

**Affiliations:** 1 Discipline of Pathology, Faculdade de Medicina do ABC, Santo André, SP, Brazil; 2 Instituto Médico Legal, São Paulo, SP, Brazil

**Keywords:** testosterone, estradiol, adipose tissue, postmenopause, testosterona, estradiol, tecido adiposo, pós-menopausa

## Abstract

**Objective** The present article aims to evaluate the impact of testosterone treatment on the expansion of visceral, subcutaneous and intramedullary adipose tissue of ovariectomized rats and the visceral and subcutaneous fat expression of peroxisome proliferator-activated receptors (PPARs) gamma*.*

**Methods** In total 48 female Wistar rats were castrated and randomly divided into 6 treatment groups: group E2 was submitted to estradiol 5 μg/day; group T, to testosterone 5 μg/day; group E2 + T, to estradiol 5 μg/day + testosterone 5 μg/day; group TT, to testosterone 30 μg/day; group E2 + TT, to estradiol 5 μg/day + testosterone 30 μg/day; and placebo was administered to group P. After 5 weeks, the rats were euthanized, the inguinal and visceral adipose tissues were harvested, weighted, and had their PPAR gamma expression evaluated by reverse transcription quantitative polymerase chain reaction (RT-qPCR). The right femurs were harvested and histologically prepared to perform the number count of the intramedullary adipocytes.

**Results** The expansion of visceral fat tissue was much higher in the TT group when compared with other treated groups (*p* < 0.001). The TT group also showed a higher expansion of inguinal fat (*p* < 0.01), and groups E2 + T and E2 + TT presented lower growth compared to the P group (*p* < 0.01). The number of femur intramedullary adipocytes only showed significant differences between groups TT and E2 + TT (*p* < 0.05). The expression of PPAR gamma showed no differences among the groups.

**Conclusion** The use of testosterone in high doses leads to an important expansion in both visceral and inguinal adipose tissues. Association with estradiol exerts an expansion-repressive effect on the visceral and inguinal adipose tissues.

## Resumo

**Objetivo** Este artigo tem como objetivo avaliar o impacto do tratamento com testosterona na expansão dos tecidos adiposos visceral, subcutâneo e intramedular de ratas ovariectomizadas e a expressão de receptores ativados por proliferadores de peroxissoma (RAPPs) gama nas gorduras visceral e subcutânea.

**Métodos** No total, 48 ratas Wistar foram castradas e divididas aleatoriamente em 6 grupos de tratamento: o grupo E2 recebeu estradiol 5 μg/dia; o grupo T, testosterona 5 μg/dia; o grupo E2 + T, estradiol 5 μg/dia + testosterona 5 μg/dia; o grupo TT, testosterona 30 μg/dia; o grupo E2 + TT, estradiol 5 μg/dia + testosterona 30 μg/dia; e o grupo P recebeu placebo. Após 5 semanas, as ratas foram submetidas a eutanásia, o tecido adiposo inguinal e visceral foi coletado, pesado, e se avaliou a expressão dos RAPP gama por reação em cadeia da polimerase via transcriptase reversa quantitativa (RCP-TRq). Os ossos do fêmur direito foram colhidos e processados histologicamente para contagem de números de adipócitos intramedulares.

**Resultados** A expansão do tecido adiposo visceral foi muito maior no grupo TT quando comparado a outros grupos tratados (*p* < 0,001). O grupo TT também apresentou maior expansão da gordura inguinal (*p* < 0,01), e os grupos E2 + T e E2 + TT apresentaram menor crescimento em relação ao grupo P (*p* < 0,01). O número de adipócitos intramedulares no fêmur mostrou apenas diferenças significativas entre os grupos TT e E2 + TT (*p* < 0,05). A expressão de RAPP gama não mostrou diferenças entre os grupos.

**Conclusão** O uso de testosterona em altas doses leva a uma importante expansão nos tecidos adiposos visceral e inguinal. A associação com o estradiol exerce um efeito repressivo de expansão nos tecidos adiposos visceral e inguinal.

## Introduction

Sexual hormones are involved in the balance of energy, and they play essential roles in the control of food intake, energy metabolism and body weight.^1^ Body changes observed during the transition to menopause and in the postmenopausal period, with increasing body weight and a different pattern of fat distribution (transfer of the main fat storage from the femoral-gluteal region to the abdominal region) are examples of this involvement.^2^ ^3^

The cell mechanisms implicated in this kind of change are not yet completely clear.^4^ ^5^ What has been revealed is that estrogen affects energy metabolism in a genomic manner via the estrogen receptor (ER) or G protein-coupled estrogen receptors (GPERs).^6^ Modulation of these receptors determines the action of anti-lipogens, the increase in insulin sensitivity, glucose tolerance, and the decrease in body weight and visceral mass.^1^ ^6^ Androgen receptors (ARs) are also present in the fat tissue, but there is less evidence on their effect.^7^ ^8^ Some evidences associate androgens to lipogenesis stimulation and lipolysis inhibition on white visceral fat.^1^ ^8^

Estrogen replacement during the postmenopausal period is associated with the reduction in visceral fat, the distribution of android fat mass and lower body mass index (BMI).^9^ ^10^ These effects determine a favorable metabolic profile with less risk of diabetes and mortality.^11^ ^12^

Different from estrogen, the impact resulting from androgen replacement on fat tissue is less often applied. Few clinical trials,^8^ ^13^ ^14^ most of them using heterogeneous methods, have evaluated the outcomes of this therapy among women during the postmenopausal period.

Some concerns regarding the impacts of androgen replacement therapy in women's health consider that supraphysiological doses may determine an inflammatory response on the fat tissue. Such response, especially at a visceral site, is associated with diseases such as resistance to insulin, dyslipidemia, diabetes, cardiovascular diseases and stroke.^1^ ^6^

The presents paper has the aim of studying the effects on fat tissue of doses of physiological and supraphysiological testosterone associated or nor with estradiol. The authors aim to show that high doses of testosterone define an unhealthy expansion of the visceral, subcutaneous and intramedullary fat tissues.

## Methods

### Trial Design

In total 48 Wistar female rats (*Rattus norvegicus albinus*) were offered and cared for by the animal research facility of Faculdade de Medicina do ABC, Brazil. The animals were fed using Nuvilab CR1 (NuVital Health, Long Beach, NY, US) and water “ad libitum,” properly filtered in a feeding bottle. Artificial lighting was controlled to obtain light/dark cycles of 12 hours each, and temperature between 20 and 28°C, between 60% and 85% of air exchange/hour.

The animals underwent bilateral ovariectomy surgeries (OVXs). After the OVX, the animals underwent vaginal colpocytology on a daily basis for two months. Colpocytology was used to assess the cessation of the estrous cycle to evaluate the possibility of hypoestrogenism.

To confirm the possibility of hypoestrogenism, colpocytology was considered in the diestrus phase for five days in a row.

Then, the animals were randomly divided into 6 groups consisting of 8 animals each. Each group underwent the following hormone treatment: group P: placebo; group E2 + T: estradiol (E2) 5 μg/female rat/day + testosterone 5 μg/female rat/day; group T: testosterone 5 μg/female rat/day; group E2 + TT: estradiol (E2) 5 μg/female rat/day + testosterone 30 μg/female rat/day; group TT: testosterone 30 μg/female rat/day; and group E2: estradiol (E2) 5 μg/female rat/day.

The dose of testosterone dose was calculated to be 6 times higher than that of estradiol. The rationality on this is that most commercial estradiol transdermal patches for women release 50 mcg/day, and the only testosterone transdermal patch ever marketed (Intrinsa, Warner Chilcott UK Ltda, Milbrook, Larne, UK) releases 300 mcg/day (6 times lower).

During 5 weeks, each group was submitted to a corresponding hormone dose based on the daily volume of 0,1 mL, which was applied by subcutaneous injections in the dorsal region. The hormones were prepared and then diluted in sesame oil, Sesame oil was also used by itself as a placebo.

The entire experiment was approved by the Animal Experimentation Ethics Committee of Faculdade de Medicina do ABC (CEUA-FMAB, in Portuguese), under number 01/2016.

### Material Collection

A few moments before the euthanasia, a blood sample was collected for glycaemia tests. After the euthanasia, the animals had their right femurs collected, as well as their visceral adipose and inguinal tissues. The femurs were kept in a 10% formaldehyde solution for the histological and morphometric analyses. Adipose tissues were weighted and stored in – 80°C for future analysis of peroxisome proliferator-activated receptors (PPARs) gamma data using the real-time polymerase chain reaction (PCR) technique.

### Histology

The femurs were fixed with 10% formaldehyde during 24 hours, and then they were decalcified in a 7% ethylenediaminetetraacetic acid (EDTA) solution with 2% paraformaldehyde in a 0.1-M phosphate buffer (pH 7.4) during 160 days at room temperature. The samples were dehydrated in graded concentrations of ethanol, and then they were embedded in paraffin. Serial 7-μm sections were made using a manual Leica RM-2245 (Leica Biosystems, Nussloch, Germany) microtome, and they were stained with hematoxylin and eosin (H&E).

### Morphometry

The morphometric analysis for the estimation of the volume density (Vv) of the intramedullary adipocytes selected five photomicrographs of each group at a magnification of 100X . For the evaluation of the adipocyte Vv, crosshairs of points superimposed on photomicrographs were also needed, and the relation proposed by Weibel was used:^15^

Vv = P1/P, where
Vv = volume density of a given component;
P1 = number of incident points on the component studied;
P = total of incident points on the volume unit

### PPAR Gamma Gene Expression RT-qPCR

The total RNA was extracted from ~ 1 cm^2^ of adipose tissue using QIAzol lysis reagent (Qiagen, Hilden, Germany). The amount of RNA was determined using NanoDrope (Thermo Scientific, Waltham, MA, US) spectroscopy, and diluted to a final concentration of 50 ηg/μl in 20 μl. In total, 1 μl of RNA was used for the synthesis and amplification of complementary DNA (cDNA), which followed the protocol of the high-capacity RNA-to-cDNA kit (Applied Biosystems, Foster City, CA, US). Reverse transcription quantitative polymerase chain reaction (RT-qPCR) was performed using the PPAR gamma gene (*Mm00440940_m1*), and the endogenous control GAPDH (*Mm99999915_g1*) followed the TaqMan Universal PCR Master Mix Kit (Applied Biosystems) using the StepOne Real-Time PCR System (Life Technologies, Foster City, CA, US). Real-time PCR reactions were conducted as follows: after a pre-denaturation and polymerase-activation program (2 minutes at 50°C and 10 minutes at 95°C), 50 cycles, each one consisting of 95°C for 15 seconds and of 60°C for 1 minute. The negative controls consisted of wells in which the cDNA was absent. The relative expression of PPAR gamma/GAPDH was calculated using the equation ΔCt, which expresses the difference between the number of threshold cycles (Cts) of the target genes and the endogenous control.

### Statistical Analysis

The results were calculated and analyzed by one-way analysis of variance (ANOVA) test and the Tukey Test using the GraphPad Prism 5 (GraphPad Software, Inc., San Diego, CA, US) software. Results ≤ 0.05 (*p* < 0.05) were considered relevant.

## Results

By the end of the fifth week of treatment, we noticed that the average body weight was different among the groups (*p* = 0.018), considering that group TT reached a higher final average weight of 307 ± 11.8 g, and group E2 + TT presented the lowest average, 264 ± 6.9 g (Table 1).

**Table 1:** Comparison of the final weight and the visceral and inguinal fat of the study groups

	Groups (mean ± standard error of the mean)						
	P	E2	T	E2 + T	TT	E2 +TT	*p*-value
Final weight (g)	291 ± 10.1	273 ± 5.3	271 ± 8.6	284 ± 8.5	307 ± 11.8	264.7 ± 6.9	0.018
Visceral fat (g)	6.5 ± 0.7	5.3 ± 0.5	3.8 ± 0.3	3.9 ± 0.7	10.3 ± 1.2*	4.5 ± 0.7	< 0.01
Inguinal fat (g)	2.6 ± 0.22***	2 ± 0.22	1.7 ± 0.13	1.4 ± 0.17	3.1 ± 0.2**	1.4 ± 0.13	< 0.01

Abbreviations: group E2, estradiol 5 μg/day; group E2 + T, estradiol 5 μg/day + testosterone 5 μg/day; group E2 + TT, estradiol 5 μg/day + testosterone 30 μg/day; group T, testosterone 5 μg/day; group TT, testosterone 30 μg/day; group P, placebo.

Notes: Tukey test: * = *p* < 0.05 (TT versus E2, T, E2 + T e E2 + TT); ** = *p* < 0.05 (TT versus T, E2 + T e E2 + TT); *** = *p* < 0.05 (P versus E2 + T e E2 + TT).

**Fig. 1:**
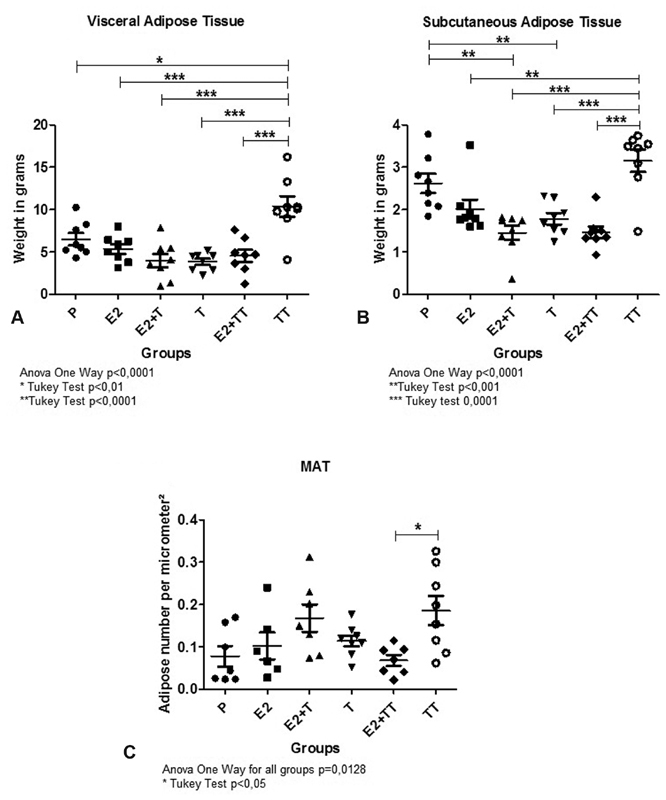
(**A**) Weight of the visceral and (**B**) subcutaneous fat tissues of all treatment groups. (**C**) Distribution of the number of intramedullary adipocytes in all treatment groups. * Represents the results with significance, that is, *p* < 0.05.

**Fig. 2:**
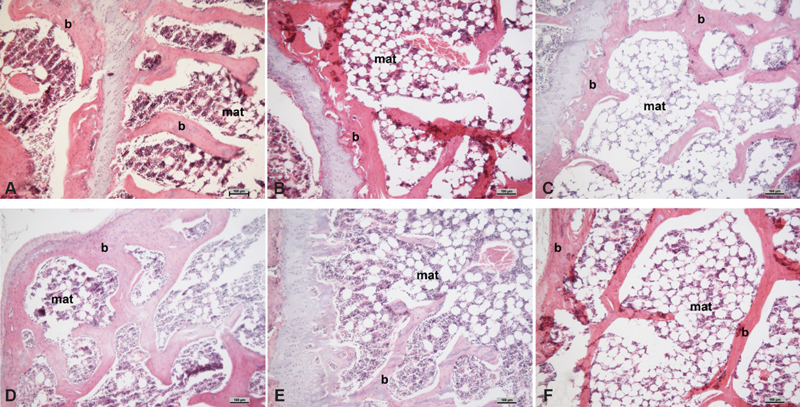
Photomicrograph of the tibiae (b) with marrow adipose tissue (MAT) stained with hematoxylin and eosin (H&E). (**A**) Group P; (**B**) group E2 + T; (**C**) group T; (**D**) group E2 + TT; (**E**) group TT; (**F**) group E2.

The average visceral fat weight was different among groups (*p* < 0.0001), and was observed to be much higher in group TT when compared with the other groups. Nevertheless, regarding inguinal fat, the only group with a higher average weight than that of group TT was group P. On the other hand, the groups that have were submitted to both estrogen and testosterone presented an average weight in this particular fat region that was considerably smaller than that of group P (*p* < 0.001), as seen in Fig. 1A and B. By using the micrometer, the number of adipocytes expressed in the intramedullary region was shown to differ among groups (*p* = 0.012), presenting a significant difference between groups TT and E2 + TT (*p* < 0.05) (Fig. 1C and Fig. 2).

**Fig. 3:**
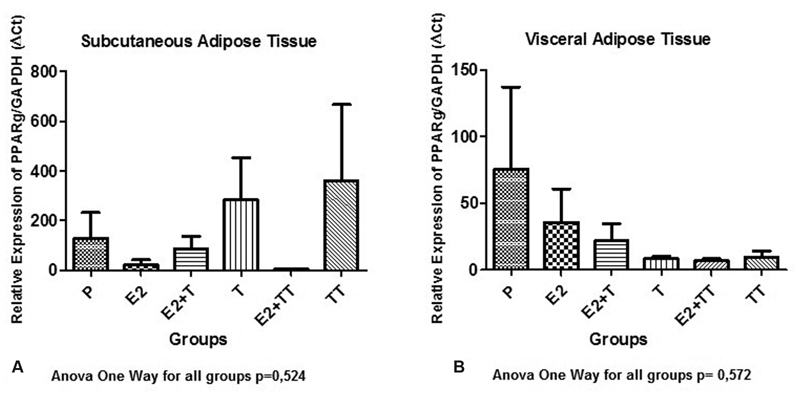
Expression of the peroxisome proliferator-activated receptor (PPAR) gamma gene in the subcutaneous and visceral adipose tissues in all treatment groups.

The PPAR gamma data did now show any statistical difference among the groups in any of the adipose tissues analyzed. Despite that, a different behavior was noticed in terms of data amongt tissues: regarding the subcutaneous tissues, the groups submitted to isolated testosterone doses presented a higher average number than group; P regarding the visceral tissue, all groups presented lower numbers than group P (Fig. 3A and B).

## Discussion

The present study assessed the impact of the treatment on visceral, subcutaneous and intramedullary fat tissues in 30-week-old female ovariectomized rats.

Two daily doses of testosterone were applied. One of the doses contained an equivalent volume of estradiol (5 μg/day), and the other dose contained a volume of estradiol 6 times higher (30 μg/day) for over 30 days. The group treated with high doses of testosterone showed a relevant visceral and subcutaneous fat growth in comparison to the other groups, which is something that contributed to a heavier final weight in this group. In a different manner, the group submitted to the lower dose showed fat growth similar to the control groups (placebo and isolated estradiol).

The testosterone serum levels show a direct link with the growth in visceral fat tissue in women going through postmenopause.^16^ ^17^ ^18^ However, there are few studies assessing the effects of androgen replacement in fat tissue during this period of a woman's life.^13^ ^14^ ^16^ ^17^ ^18^ ^19^ ^20^ Despite applying a heterogeneous method, evidence confirms the idea that postmenopausal women using androgen have increased lean mass and visceral fat.^1^ ^3^ ^14^ ^19^ ^20^ ^21^ ^22^

Androgen supplementation in animals has been observed to increase visceral fat.^23^ ^24^ ^25^ There is a hypothesis that the estrogen serum level influence on this effect.^24^ Iwasa et al^25^ have evaluated such effect in female rats which have undergone ovariectomy regarding the chronic doses of testosterone (associated or not to estradiol) and its link to food intake, body weight and white fat weight.

Results found by Iwasa et al^25^ are conflicting with our results. In comparison with groups treated with testosterone or not and with no association with estrogen, Iwasa et al^25^ observed a relevant reduction in the final weight, as well as in the weight of visceral and subcutaneous fat in the group treated with testosterone. Their study concluded that testosterone, once used apart from other elements, had an inhibitory effect on weight gain and adiposity. This conclusion was not in line with the results of the group treated with high doses of testosterone in our study, since the high dose determined a considerable increase in adiposity. Moreover, the group submitted to low doses of testosterone did now show any differences when compared with the control groups.

Iwasa et al^25^ also observed that the association of testosterone to estrogen in ovariectomized female rats determined an increase in body weight and in the weight of visceral and subcutaneous fat. Their study concluded that testosterone lowered the inhibitory effect of estrogen on body weight and adiposity. Despite that, our study, using a different method, showed that the groups treated with estrogen had similar gains regarding weight and adiposity, independent of testosterone. However, it is worth nothing that the groups treated with both kinds of hormone have shown a relevant lower subcutaneous fat weight in comparison to group P, which is something that did not occur with the group treated with isolated estrogen.^25^

The difference in results of the study by Iwasa et al^25^ and our study can be explained based on the period of treatment (35 days *versus* 16 days respectively) and the testosterone doses used. Considering the results from both studies, it can be said that high doses of testosterone can stimulate excessive adipogenesis, while low doses in association with estradiol can determine its inhibition.

The intramedullary fat tissue represents 70% of the total volume of bone marrow in a healthy young adult,^26^ being the third largest fat storage in the human body. The percentage of its contribution to the total volume of body adipose tissue may vary from 1% to 30%.^26^ ^27^ ^28^ Such tissue presents structural characteristics and lineage specific traits, which suggests that marrow adipose tissue (MAT) adipocytes have a source different from that of white and brown fat.^28^ Therefore, it is interesting to observe any possible impacts resulting from the testosterone treatment on this fat tissue.

When assessing the number of adipocytes in the bone marrow (which represents the amount of fat in this region), an increase in the average number of adipocytes could be noticed in the group treated with a high dose of testosterone, despite the difference not being statistically relevant in relation to the control groups. Thereby, this fat tissue site showed a behavior pattern similar to that of other fat sites when treated with sex hormones.

It is worth saying, though, that the number of adipocytes was calculated based on the entire femurs, and did not reveal any differences between the proximal and distal regions. Such information is relevant when considering that there may be two forms of MAT: variable (regulated - rMAT) and constant (constitutive - cMAT). The difference between them is based on the bone marrow region and on the different response to external stimuli. Besides, there are different kinds of development pattern, adipocyte size, lipid saturation, and expression of transcription factors. The rMAT form is found inside the red marrow (proximal region of the skeleton), and contains more saturated fat and has a higher sensibility to external stimuli. The cMAT, on the other hand, is located inside the yellow marrow (distal region of the skeleton), and presents more resistance to external stimuli.^27^

The number of intramedullary adipocytes was calculated, and it was diffused in most groups, reducing the possibility to determine the difference between them. Besides that, there is evidence that ovariectomized rats have an increase in MAT,^27^ ^28^ something not showed in our study. The accuracy of the methodology for the histological evaluation of the adipocytes was questioned,^29^ and could be the reason for such an incoherent result. The coloration using osmium tetroxide and further analysis using micro computed tomography (micro-CT) is considered the standard procedure.^29^ However, the risks of the toxicity related to osmium tetroxide and the lack of a microtomograph kept the team from applying such methods.

The PPARs influence the gene network expression involved in adipogenesis, lipidic metabolism, inflammation, and the maintenance of metabolic homeostasis. The PPAR gamma specifically acts as a regulator on adipogenic media, being considered a fundamental regulator for adipocyte differentiation.^30^

Estrogen has an impact on the transcriptional activity of PPRA gamma, and it inhibits its effect on adipocyte differentiation.^31^ On the other hand, the effect of androgen over the activity of PPAR gamma on the fat tissue has not been determined. Compatible to what is mentioned in the technical literature, the groups treated with estrogen in the present study revealed a reduced PPAR gamma expression when compared with the control groups in both assessed fat tissues.^31^

The pattern of expression of PPRA gamma varied according to the location of the fat in the groups treated with testosterone. Regarding subcutaneous fat, despite not being statistically significant, the expression was higher than that of the control groups. When considering visceral fat, it was lower in both groups.

Considering that PPRA gamma is involved in adipogenesis, it is expected that its expression would be higher in those groups with higher adipogenesis.^30^ This occurred in subcutaneous fat, and the TT group presented a higher fat growth as well as the highest expression. Nevertheless, this did not occur in visceral fat, in which the PPRA gamma expression was inhibited. Dysfunctional visceral adipose expansion results in an inflammatory state and increases the release of inflammatory cytokines and free fatty acids,^32^ which worked as an inhibitor to the activity of PPRA gamma.^33^ ^34^

The present study has some limitations. An individual assessment of each region of the bone marrow could determine different results regarding intramedullary adipose tissue, but this was not possible due to technical reasons. Moreover, the coloration of the bone marrow using osmium tetroxide and further analysis using micro-CT is considered to be the standard procedure for MAT analysis.^29^ However, the risks of the toxicity related to osmium tetroxide and the lack of a microtomograph kept the team from applying such methods in the present study.

## Conclusion

High doses of testosterone replacement in OVX rats lead to an expansion of visceral, subcutaneous and bone marrow fat. This phenomenon seems to be abrogated by estradiol replacement. The increase in visceral fat is not linked to an increased PPAR gamma expression.
